# Metagenome sequence data mining for viral interaction studies: Review on progress and prospects

**DOI:** 10.1016/j.virusres.2024.199450

**Published:** 2024-08-21

**Authors:** Mohammadreza Rahimian, Bahman Panahi

**Affiliations:** a Department of Biology, Faculty of Basic Sciences, University of Maragheh, Maragheh, Iran; b Department of Genomics, Branch for Northwest & West Region, Agricultural Biotechnology Research Institute of Iran (ABRII), Agricultural Research, Education and Extension Organization (AREEO), Tabriz, Iran

**Keywords:** NGS, Identification, Mining, Metagenomic data

## Abstract

Metagenomics has been greatly accelerated by the development of next-generation sequencing (NGS) technologies, which allow scientists to discover and describe novel microorganisms without the need for conventional culture techniques. Examining integrative bioinformatics methods used in viral interaction research, this study highlights metagenomic data from various contexts. Accurate viral identification depends on high-purity genetic material extraction, appropriate NGS platform selection, and sophisticated bioinformatics tools like VirPipe and VirFinder. The efficiency and precision of metagenomic analysis are further improved with the advent of AI-based techniques. The diversity and dynamics of viral communities are demonstrated by case studies from a variety of environments, emphasizing the seasonal and geographical variations that influence viral populations. In addition to speeding up the discovery of new viruses, metagenomics offers thorough understanding of virus-host interactions and their ecological effects. This review provides a promising framework for comprehending the complexity of viral communities and their interactions with hosts, highlighting the transformational potential of metagenomics and bioinformatics in viral research.

## Highlights

• Examining integrative bioinformatics methods used in viral interaction research, this study highlights metagenomic data from various contexts.
• Accurate viral identification depends on high-purity genetic material extraction, appropriate NGS platform selection, and sophisticated bioinformatics tools like VirPipe and VirFinder.
• The diversity and dynamics of viral communities are demonstrated by case studies from a variety of environments.
• In addition to speeding up the discovery of new viruses, metagenomics offers thorough understanding of virus-host interactions and their ecological effects.

## 1 Introduction

Metagenomics is a biotechnological field that has advanced with the progression and emergence of cutting-edge NGS technologies. Companioning metagenomics with NGS enables researchers to identify and characterize novel microorganisms without using traditional laboratory processes. This companion can identify hard-to-culture microorganisms from the sequenced genetic material of vast samples, such as patients, plants, and the environment (Duan et al., 2021; Kawulok et al., 2019; Yang et al., 2022). These approaches make the identification of difficult-to-cultivate virus pathogens from medical samples (Qian et al., 2023), unveiling virus community (mostly bacteriophages due to their high abundancy among DNA viruses) of virome (Eissler et al., 2020), and discovery of any interaction between host and virus (Chow et al., 2015) much more applicable, easier, and faster.

The first and most crucial step to performing successful metagenomics analysis is achieving high-purity genetic material with optimum concentration. Choosing the best method to extract genetic material based on the sample type is crucial to achieving such genetic material. Nowadays, researchers generally utilize commercial kits to extract and purify genetic materials. According to the recently published systematic review, commercial kits produced by Qiagen are the most widely used commercial kits for genetic material extraction (Bassi et al., 2022). After the mentioned process, researchers must choose and evaluate proper NGS platforms to sequence and achieve genetic material data (Patel et al., 2022).

In the second stage, bioinformatics tools analyze the raw data and perform quality control. The assembly software creates contigs, or expanded sequences, by reconstructing the short meta-genome information during the assembly process. Once metagenomic sequencing data has been assembled into contigs, the next stage is to align these contigs with the original microbial genomes from which they originated. To rebuild each genome found in a complicated metagenomic sample, a procedure known as "binning" is essential (Andrade-Martínez et al., 2022).

Following the binning procedure, the sequences were examined utilizing the alignment approach with tools linked to bioinformatics, such as BLAST (Patel et al., 2022), virus identifiers (Ren et al., 2017), or automatic virus identification pipelines (Kim et al., 2023a). In the last step, after phylogenetic analysis, a phylogenetic tree based on analyzed data is depicted (Patel et al., 2022). It is important to note that many other tools can be used in bioinformatics, such as assembly tools, organizing tools, and so on, depending on the research goals.

## 2 Virus discovery

**Fig. 1:**
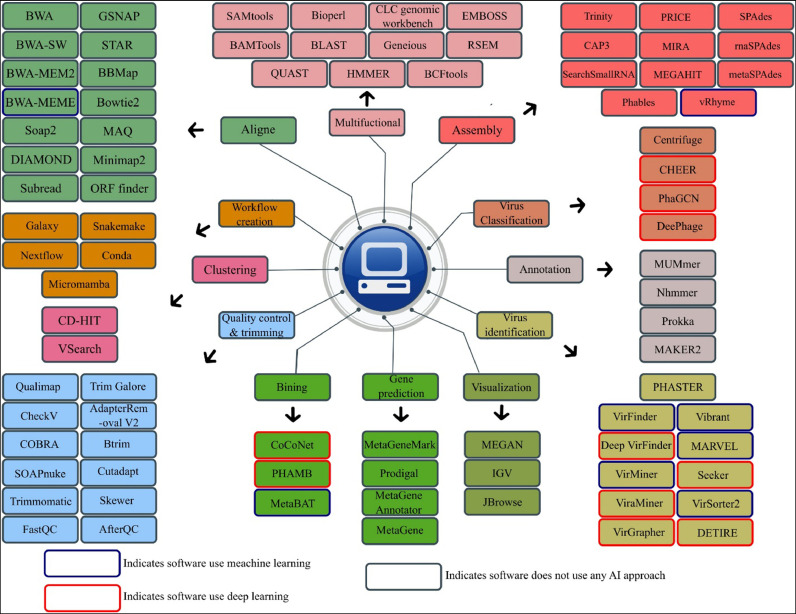
Computational tools for virus discovery from metagenome data.

The discovery of viruses via traditional laboratory is very time-consuming, and it is mainly impossible to identify the vast community of viruses owing to their abundance. The development of virus discovery through metagenomics makes virus discovery much faster than before and has become a promising approach for identifying viruses. However, even with the use of metagenomics, there are still challenges that researchers must face. A notable challenge in this manner is the high diversity of virus genomes compared with the source genomes. This diversity complicates identifying and assembling target virus genomes due to the difficulty of identifying unknown virus genomes with high dissimilarity compared with known viruses in bulk data originating from the source genome. Reading and analyzing such bulk data can also be challenging and demand sophisticated tools (Smits et al., 2015). Despite the mentioned challenges, virus discovery by metagenomics studies is a fast and popular method. Advances such as the introduction of novel sequencing technologies such as NGS and third-generation sequencing (TGS) (Satam et al., 2023), as well as innovative bioinformatics-related tools (Fig. 1) and advancements in computer sciences, like the emergence of AI, reduce the impact of the challenges and accelerate virus discovery through cutting edge pipelines with the ability to predict and quantifying contigs results in taxonomic or functional profiling of viruses (Liu et al., 2021)—the key differences between these two methods provided in Table 1.

**Table 1:** Comparing virus discovery methods: culturing vs. molecular approaches.

Differences	Method	
	Data mining	Laboratory methods
Procedure	Analyzing transcriptome big data	Isolation and genome sequencing of viruses isolated for specific host
Speed	Viruses discovered swiftly	These procedures are slow and time-consuming
Cost	It is cost-effective	More expensive than data mining
Requirement	It does not require expensive equipment, but it demands high-tech computational systems.	It requires expensive sequencing systems and expert professionals to manage the whole process.
Integration with other data	It can be integrated with other omics data, such as proteomics and metabolomics, to comprehensively understand the virus and its host interactions.	Virus features understanding demanded further analyses in the laboratory
Automation	It can be automated with the help of sophisticated bioinformatics tools and AI.	All of the analyses performed by specialists

TGS technologies (PacBio and Oxford Nanopore in particular) have become strong for replacing conventional NGS platforms, which mainly use short-read sequencing. Nanopore sequencing detects changes in electrical current as single-stranded DNA travels through a nanopore, enabling even longer readings (10,000 to 30,000 base pairs read average). In contrast. PacBio uses single-molecule real-time (SMRT) sequencing with the ability to read 10,000 to 16,000 base pairs on average. Comparing these long-read capabilities to short-read NGS, which usually yields readings of only a few hundred base pairs, reveals considerable advantages. Also, these technologies show promise in virus research since they can produce entire viral genome assemblies in a single read, which is essential for researching viral evolution, transmission, and drug resistance (Satam et al., 2023).

**Fig. 2:**
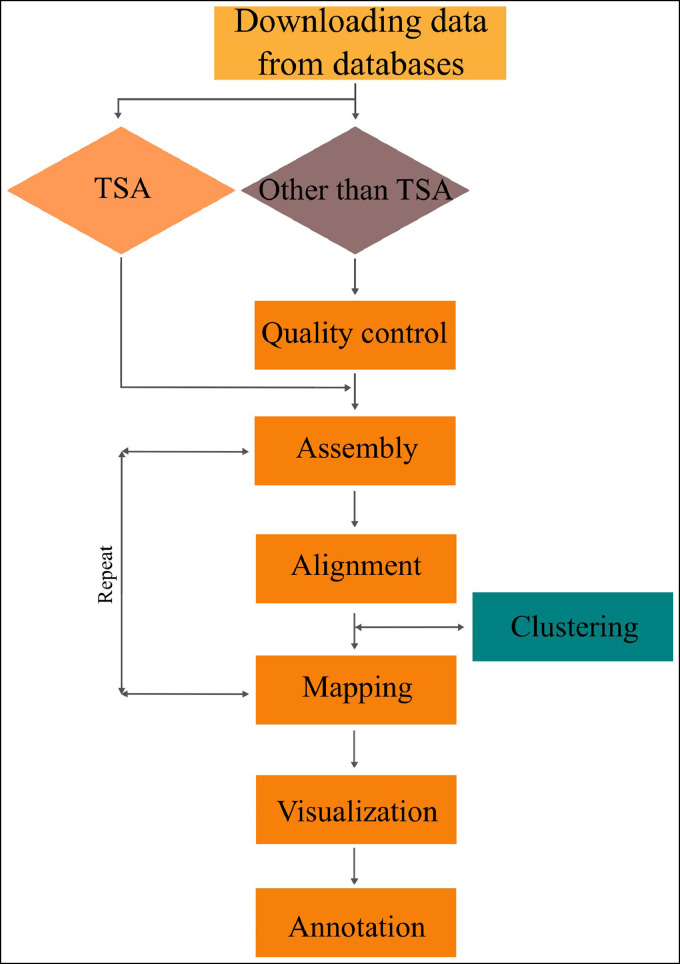
Workflow for novel virus discovery through transcriptome data mining.

Every virus identification pipeline has its pros, cons, or limitations, therefore a single pipeline cannot be suitable for all studies with different purposes (the virus discovery procedure can proceed as Fig. 2 with the differences in the type of tools and data) (Odom et al., 2023). This phenomenon has caused companies to compete to provide a suitable platform for studies to continue. One of the innovative pipelines introduced recently is VirPipe, which consists of three steps, including reference mapping, contig analysis, and taxonomic classification to identify new viruses from Illumina and nanopore-originated data. This method provides a customizable user, user-friendly pipeline with no cons of similar pipelines, such as limitation of analysis numbers, downloading extensive data, and manually installing all required programs (Kim et al., 2023a).

Moreover, researchers have recently created or updated several pipelines with the potential to unravel unknown virus identification issues. virMine is a three-step virus identification pipeline that can read short and long sequences, assemble genomes, and predict viruses. This software is notable for its capability to perform quality control after assembly, its three assembly methods, and its potential to predict both viruses and prophages in the contigs (Johnson and Putonti, 2022).

Researchers can utilize AI in transcriptomics studies, as they do in many other biological fields. AI plays a crucial role in accelerating transcriptome studies and enhancing their accuracy (Panahi and Shahi, 2024). Moreover, according to the previous investigation, the ML approach can successfully predict viral contigs in sequencing origin data (Bzhalava et al., 2018). Successful utilization of ML in virus identification persuades researchers to investigate the application of novel AI-based tools such as deep learning (DL) in virus identification. Such investigations proved that DL methods are reliable for predicting viral genomes from source genome data (Dasari and Bhukya, 2022). In this case, companies developed many popular AI-related tools based on ML (e.g., VirFinder (Ren et al., 2017)) and DL (e.g., DeepVirFinder and PPR-Meta) to assist researchers in identifying viruses in metagenomics data sets (Ho et al., 2023).

Companies have developed new AI-based virus identification tools that have performed better in recent years. For instance, Miao et al. recently developed the DETIRE tool, a hybrid deep-learning model appropriate for viral genome identification from metagenome data. As a notable rival of CHEER, DeepVirFinder, and PPR-Meta, this tool can read short sequences more than the mentioned tools (Miao et al., 2023). Verifier is another DL-based tool with good potential in reading short sequences. Scrutinizing sample studies indicated that this tool has better short-sequence reading than the three methods mentioned (Miao et al., 2022). Besides many benefits, there are still some challenges in utilizing the mentioned tools. Due to the inability of these tools to read long sequences, the tool developers program them to turn long sequences into numerous short sequences. However, a recent tool called VirGrapher makes reading long sequences possible. The creators of VirGrapher designed it to read long sequences by utilizing an innovative AI platform called graph convolutional network (GCN). This platform can consider long sequences as a graph and train itself to investigate any possible connection of the nodes of that graph, resulting in the ability to read the long sequence. These AI-based tools show better accuracy, recall, precision, specificity, and F1 score than CHEER, DeepVirFinder, and PPR-Meta, making VirGrapher a reliable tool for identifying viruses from bulk sequence data (Miao et al., 2024).

**Fig. 3:**
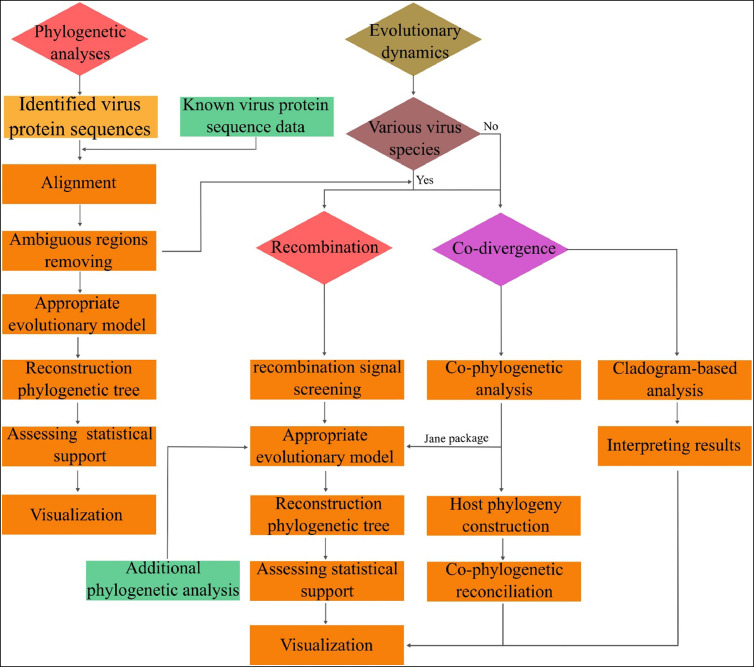
Flow chart of phylogenetic analysis and evolutionary dynamics examination methods.

Researchers can assess gene prediction in parallel with virus identification, as this preliminary data serves as crucial input for functional annotation and taxonomic classification. Functional annotation can be performed by aligning the predicted ORFs against Clusters of Orthologous Groups of proteins (COG), Pfam (Jaiani et al., 2020), EggNOG, nr, SwissProt, and KEGG databases while by performing BLASTP against nr and SwissProt and analyzing output in MEGAN software taxonomic classification can be executed (Le et al., 2022). Also, phylogenetic analyses are another evaluation of metagenomics-derived data, performed similarly to the workflow provided in Fig. 3.

Various studies have proved the high potential of metagenomics approaches in discovering viruses. One of the proper examples of a metagenomics study in virus identification based on leveraging different seawater (hydrothermal) samples is the study conducted by Cheng et al. The study identified 1731 complete viral genomes via VirSorter and VIBRANT tools. These viral genomes belong to ssDNA viruses including *Microviridae*, CRESS group, and *Inoviridae*, dsDNA phages such as *Myoviridae, Siphoviridae, Podoviridae, Herelleviridae, Ackermannviridae*, and unclassified groups in *Caudovirales* phage class, and nucleocytoplasmic large DNA viruses (NCLDVs) like *Iridoviridae, Lavidaviridae, Marseilleviridae, Mimiviridae, Phycodnaviridae, Pithoviridae*, and unclassified NCLDV class showcasing divergent virus community of the target environment (Cheng et al., 2022). The results of a different investigation showed a large seasonal and regional variability in viral populations in the Black Sea. The *Myoviridae, Podoviridae*, and *Caudovirales* are the three main viral families. Notably, a sizable fraction of the viral genes are still unknown, suggesting a significant amount of unknown viral diversity in this area (Jaiani et al., 2020).

In Gonio, the predominant viral species in May include phages of *Synechococcus, Pelagibacter*, and *Puniceispirillum*, together with algal viruses including *Bathycoccus and Emiliania huxleyi* infectious viruses. By September, there was a significant change in the viral community makeup, with 94 % of the community composed of *Synechococcus* phages. Similar seasonal trends are seen in Poti, where *Synechococcus* phages predominate after *Pelagibacter* by September, and *Synechococcus* phages are common in May (Jaiani et al., 2020). On the other hand, the South China water viromes (SCSV) data set, which consists of 122 Gb of sequences, provides an extensive perspective on viral diversity at varying water depths. Researchers used VirSorter and IMG/VR 2.0 datasets to predict 121,952 viral contigs. While other studies focused on unveiling virus families, the SCSV study focused on the prevalence of identified viruses in surface and deep sea and their sources. According to the community structures and comparisons of the surface and deep sea viromes in the SCSV, several potential sources of the deep sea viromes were hypothesized in the study (Liang et al., 2019).

First, certain viruses are present throughout the water column in proportion to their host species. For example, surface and deep-sea waters were loaded with phage HTVC010P and its host. Second, the creation of deep-sea viromes may be facilitated by sedimentation mechanisms. The detection of potential autotrophic *cyanobacteria* phages in the deep sea viromes, such as Cyanophages and *Prochlorococcus* as a potential host, suggests that these viruses may descend from the euphotic zone to the deep sea alongside their host cells, which seems justifiable by sedimentation mechanisms. Thirdly, a considerable portion of viruses seem to be native to the deep sea viromes, most likely infecting the dominating species that live in the deep sea. For instance, deep-sea viromes contain viral operational taxonomic units (vOTUs) linked to *Mycobacterium* phages, which may infect *Mycobacterium* species frequently found in deep-sea habitats. Finally, the similarity of viromes across several sampling sites implies that physical transportations, both vertical and horizontal, may play a role in the creation of deep-sea viromes. Viral populations may be able to trace water sources. This theory may be confirmed by further thorough sampling and research in regions between the northeast South China Sea and the Northwestern Pacific Ocean (Liang et al., 2019).

A comparison of these studies provides some important information. The studies mentioned above highlight the dominance of particular viral families and the significance of seasonal and regional elements in forming viral communities. The approaches, meanwhile, are very different. The Black Sea study uses alignment methods, whereas the other two papers show how metagenomic approaches have evolved by using sophisticated bioinformatics tools like VirSorter, VIBRANT, and IMG/VR. In conclusion, metagenomics has greatly sped up the process of viral discovery, offering a more effective and all-encompassing strategy. In contrast, conventional laboratory approaches are laborious and have a narrow focus. The complexity of interpreting large amounts of data and the high diversity of virus genomes provide difficulties; however, advances in NGS technologies, bioinformatics tools, and AI have significantly reduced these barriers. Creating cutting-edge pipelines like VirPipe and AI-based tools like DETIRE and VirGrapher has significantly improved the accuracy and efficiency of virus identification. Comparative analyses of viral populations in various settings highlight metagenomics' capability to reveal the wide and diverse world of viruses and demonstrate the method's resilience and adaptability.

The dynamics of ecosystems and human health are significantly shaped by the intricate and diverse phenomena known as the interaction between viruses and their hosts. Numerous hosts, such as bacteria, protists, and humans, get infected by viruses, and these interactions have important ramifications for both the virus and the host. A virus's relationship with its host is essential to its spread and reproduction. Viruses must control the host's metabolic functions to multiply and produce new viral particles. This usually means modifying the host's cellular pathways, which might change gene activation, protein production, and metabolism (Samy et al., 2022; Xiao et al., 2017). Furthermore, due to the constant struggle between viruses and their hosts, which results in the formation of novel viral strains and the development of host defense systems, viral evolution and adaptability are closely linked to host-virus interactions (Ríos-Marco et al., 2016).

**Fig. 4:**
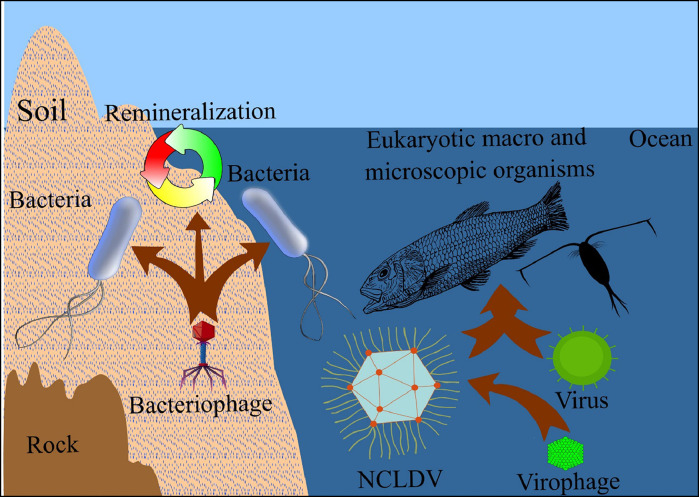
Complex VHI in the environment, including interactions between phages, NCLDVs, virophages, and other eukaryotic viruses with their hosts.

Compared to other sources outlined in the transcriptomics section, VHI has a more complicated environment (Fig. 4). The environment is thought to be a virus's ancestral nest. For instance, one significant estuary environment, the Chesapeake Bay, is home to various viruses that interact with various hosts, such as freshwater *Actinobacteria* lineages (Sakowski et al., 2021). Similarly, various viruses are present in the soil virome, interacting with microbial hosts to affect ecological processes such as the cycling of carbon and nitrogen (Duan et al., 2022). Moreover, a critical aspect of virus-host interactions is the variety of hosts that viruses can infect. Viruses have been shown to infect a wide range of hosts in marine environments, including marine heterotrophic bacteria like the OM43 clade (Buchholz et al., 2022).

In a recent study published in 2022, the effect of bacterial symbionts on nucleocytoplasmic large DNA virus (NCLDV) infections in free-living amoebae was investigated. The scientists found that in both environmental isolates and lab strains of Acanthamoeba, a particular bacterial symbiont called *Parachlamydia acanthamoebae* impeded the replication of the viennavirus. Additionally, this symbiont inhibited the growth of more complicated viruses such as tupanvirus deep ocean and *Acanthamoeba polyphaga* mimivirus. (Arthofer et al., 2022). For unveiling these complexities, Metagenomics approaches have emerged as a powerful tool for understanding the intricacies of virus-host interactions and perfectly revolutionized the VHI discovery and providing valuable insights into the structure and function of viral communities and their relationships with their hosts in various samples, especially environment samples (Okazaki et al., 2019).

## 3 Discovery of phage-bacteria interaction (PBI)

In recent years, bacteriophage research has witnessed a significant resurgence of interest, driven by the growing awareness of antibiotic resistance and the need for alternative treatments. Today, the field encompasses a broad range of topics, including phage therapy, phage ecology, phage-mediated gene transfer, and the role of phages in shaping microbial communities. The increasing availability of genomic data and advances in sequencing technologies have also facilitated the discovery of new phage lineages and the characterization of their metabolic capabilities, further expanding our understanding of these fascinating microorganisms (Plumet et al., 2022; Wittmers et al., 2022).

In addition to the influence on microbial communities, bacteriophages can impact bacterial metabolism. Bacteriophage auxiliary metabolic genes (AMGs) encode proteins that can alter the metabolic processes of the host bacterium, potentially enhancing the phage's ability to infect and replicate. Studies have demonstrated that AMGs are essential for reprogramming host metabolic pathways, including synthesizing chaperones, signaling proteins, lipid metabolism, and nitrogen metabolism. These genes play critical roles in the phage's life cycle, allowing it to adapt and thrive within its host environment (Luo et al., 2022). Phages carrying AMGs for sulfur oxidation have been extensively studied, revealing their substantial contributions to sulfur and thiosulfate oxidation in various aquatic environments, including freshwater lakes, oceans, and hydrothermal settings (Heyerhoff et al., 2022; Kieft et al., 2021).

Additionally, the diversity and distribution of AMGs in marine viruses, particularly within the Roseobacter clade, underscore their role in nucleotide biosynthesis and functional diversity, ultimately influencing the evolution of phages (Huang et al., 2021). Transferring antibiotic resistance genes (ARGs) is another important aspect of the PBI. These genes are transferred by horizontal gene transfer from phage to bacteria, spreading antibiotic resistance among bacteria (Pfeifer et al., 2022).

**Fig. 5:**
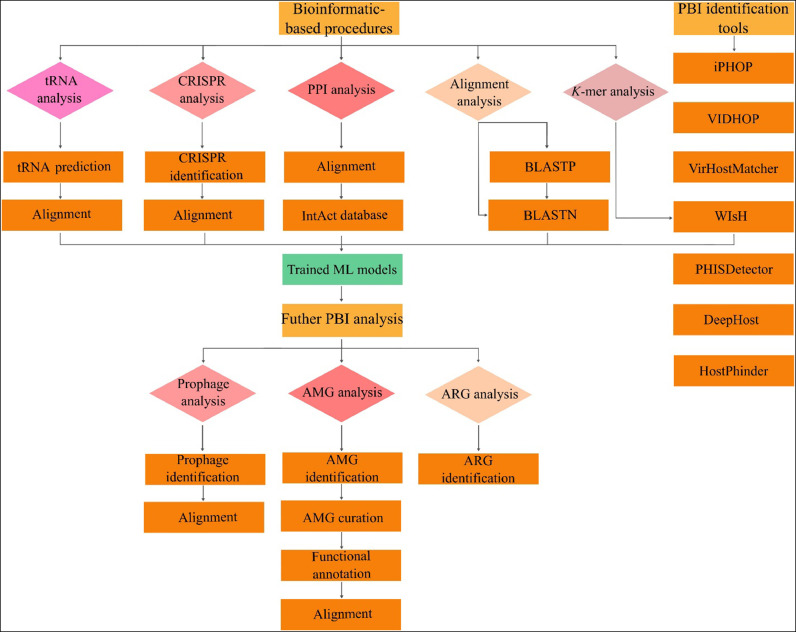
Flow chart of PBI discovery.

Scrutinizing metagenomics data gives valuable insights into PBI, including predicting bacteriophages' life cycles and hosts and identifying AMGs and ARGs. The simplest way to host the prediction of bacteriophages is using PBI discovery tools (Fig. 5), which are well designed for this cause by performing a sequence of analysis. These tools can predict the host by performing a sequence composition analysis (e.g., WIsH (Galiez et al., 2017)), utilizing DL methods (e.g., DeepHost (Ruohan et al., 2022)), and even with combination analysis including various types of bioinformatic procedure and AI-related methods (e.g., PHISDetector (Zhou et al., 2022)).

It is also possible to identify the PBI via bioinformatics techniques. Identifying and alignment of clustered, regularly interspaced short palindromic repeats (CRISPR) in identified viral contigs (Tomofuji et al., 2022) is one of the most important and prevalent techniques in this manner. While PBI discovery via CRISPR identification is used solely in relevant studies (Tomofuji et al., 2022; Trubl et al., 2021), some researchers act conservatively by using a combination of techniques, including identifying and aligning tRNA, mapping identified contigs against bacterial and archaeal genomes, and *K*-mer signature analysis of contigs via WIsH software (Du et al., 2023). It is also possible to merge the mentioned techniques with PPI analysis and unveil the networks with relevant databases such as the IntAct database. For identifying any PBI, achieved data can also be analyzed again using ML-based techniques. By implementing the mentioned procedure (except for tRNA identification), PHISDetector has reached a high detection rate (according to the type data, 0.51 at the species level and 0.73 at the family level) (Zhou et al., 2022).

AI-related methods can also be applied to PBI prediction. For instance, Wang et al. created a novel machine-learning method to forecast PB. With their approach, phage-phage, phage-bacteria (archaea), and bacteria-bacteria similarities are represented by a two-layer network. The viruses in this network are connected according to how similar their sequences are to one another; the more similar the sequences, the thicker the edge. Similarly, hosts are connected based on their sequences' similarity, with edge thickness indicating their proximity. By integrating several features, the framework predicts the possibility of contact between bacteria and phages. It starts by considering how similar the phage is to other phages known to infect the same host. Second, it assesses how similar the host is to other hosts that have contracted the same virus. Thirdly, *k*-mer frequencies evaluate the alignment-free sequence similarity between the host and phage (Wang et al., 2020).

Furthermore, consideration is given to shared CRISPR spacers between the host and phage. Finally, the method incorporates alignment-based sequence matches between the virus and host sequences. Ultimately, a network-based ML model integrates all these diverse features to predict the probability of a specific PBI occurring (Wang et al., 2020). Another example of this content is an ML-based prediction model developed by Liu et al. (2019). that developed a method for predicting virus-host associations by representing viruses and hosts as sets *V* and *H*, respectively, and defining their associations as an adjacency matrix *Y*. Positive and negative interactions between viruses and hosts are indicated by 1 and 0 in *Y*. The method begins by calculating oligonucleotide frequency (ONF) measures to determine phage-phage and host-host similarities, denoted as S*v* and S*_h_*, respectively.

Furthermore, to capture associations between hosts, the Gaussian interaction profile (GIP) kernel similarity is produced for each host. Similarity Network Fusion (SNF) combines these similarities into complete similarity networks. The model combines these commonalities and established correlations to build a heterogeneous network that links viruses and hosts. The binary matrix *Y* is then split into matrices *W* and *H* via kernelized logistic matrix factorization, which maps hosts and viruses into a common low-dimensional space. For each virus-host combination (v_i_, h_j_), the association probability p_ij_ is computed using a logistic function considering sequence similarity. Using importance weighting and neighborhood regularization improves prediction accuracy while using the AdaGrad algorithm to optimize the objective function. Metrics like AUC, AUPR, and host prediction accuracy on a benchmark dataset support the final model's ability to successfully incorporate various similarity measures and regularization strategies to predict phage-bacteria correlations with high accuracy (Liu et al., 2019).

Further PBI analysis can provide valuable information about Phage's interaction with bacteria. In the case of spreading antibiotic resistance, prophages and antibiotic resistance genes (ARGs) play a crucial role. ARGs are genetic elements that can cause antibiotic resistance, and prophages can spread these elements from one bacteria to another (Colavecchio et al., 2017). Thus, it seems prophages analysis of identified contigs consists of identification of prophages that can be done with prophage prediction tools (Table 2) and aligning them against viral DNA sequences (Zhou et al., 2022) or by investigating sequences with lysogeny-related genes such as transposases, integrases, recombinases, parAB, CI/Cro repressor, and excisionases (Yu et al., 2024); as well as identification of ARGs via related identifier tools (Du et al., 2023) cannot be neglected.

**Table 2:** Emerging tools and techniques for enhanced PBI discovery.

Performance	Tool name	Reference or link
CRISPR identification	CRISPRFinder	[176]
	PILER-CR	[177]
	CRT	[178]
	CRISPRDetect	[179]
	MinCED	https://github.com/ctSkennerton/minced
	CRISPROpenDB	https://github.com/edzuf/CrisprOpenDB
	CRISPRCasFinder	[180]
	CRISPRCasTyper	[181]
*K*-mer composition analyzing	WIsH	[164]
	VirHostMatcher	[170]
Prophage identification	Phage_Finder	[182]
	VirSorter	[183]
	DBSCAN-SWA	[184]
AMG identification	DRAM-v	[185]
ARG identification	Resfams	[186]
	AMRFinderPlus	[187]
	RGI	[188]

Another important gene that can reveal other aspects of PBI is AMG. Analysis of these genes begins with AMG identification; for insurance of the viral origin of the AMGs, a curation step must be performed. This step includes different analyses such as identifying hallmark and viral-like genes neighboring identified AMGs, checking the location of AMGs within identified contigs, identifying promoter/terminator parts of AMGs, and predicting active sites and conserved regions of a protein. The next step is functionally annotating the AMGs and aligning sequences for prediction of the host origin of the AMGs (Muscatt et al., 2023; Pratama et al., 2021; Yu et al., 2024).

The studies conducted by Yu et al., which examined PBI in seamount sediments (Yu et al., 2024), and Du et al., which investigated the built environments (Du et al., 2023), are fine examples of comprehensive PBI through metagenomics data. In seamount sediments, a comprehensive study predicted potential hosts for 1600 vOTUs using four bioinformatic approaches, including CRISPR-spacers matching, tRNA matching, nucleotide sequence homology, and *k*-mer frequencies. Three thousand nine hundred twenty-three virus-host links were found using these techniques, with tRNA matches being the most common method (3316 linkages). Even with these efforts, only sixteen percent of the vOTUs had putative hosts predicted, demonstrating the difficulty in determining host connections in various intricate contexts (Yu et al., 2024). In built environments, the prediction of virus-host linkages covered 122 vOTUs, accounting for 31 % of the genomes, using genome-spacer matches. The most frequently predicted hosts belonged to the bacterial genera *Micrococcus, Corynebacterium, Kocuria*, and *Barrientosiimonas*. However, a more extensive assembly of microbial genomes from the same dataset resulted in in situ hosts being predicted for 349 vOTUs (81 % of the viral genomes), indicating a narrow host range in these environments (Du et al., 2023).

Moreover, investigating the viral lifestyles in seamount sediments showed that almost one-third of the vOTUs were lysogenic. In the viral communities from the bulk metagenome and virome datasets, temperate viruses accounted for 27 % and 34 % of the relative abundance, respectively. This high prevalence of temperate viruses is consistent with observations from other deep-sea settings, indicating that they have a function in facilitating virus-mediated gene transfer and enabling host survival under harsh circumstances (Yu et al., 2024). Viruses with lytic cycles, on the other hand, were more common in viruses with greater virus-host abundance ratios in-built habitats, indicating that a possible rise in lytic viral infection may inhibit host growth and abundance. Conversely, more viruses with lysogenic cycles were linked to dominant hosts in most habitats, supporting the Piggyback-the-Winner hypothesis, where lysogenic viruses thrive alongside abundant hosts (Du et al., 2023).

Also, 331 putative AMGs were identified in seamount sediments, with a large proportion derived from *Proteobacteria*, consistent with *Proteobacteria* being the most frequently predicted hosts. Numerous metabolic pathways, including cofactor, carbohydrate, amino acid, vitamin, nitrogen, sulfur, and carbon metabolism, were impacted by these AMGs. Viral AMGs may have a role in biogeochemical cycles; for instance, AMGs associated with sulfur cycling (such as SELENBP1, CysK, CysH, and Sat) are essential to sulfur reduction and oxidation processes (Yu et al., 2024). A study of 99,084 protein-coding genes found in viromes in built environments showed that 38 % lacked a substantial database match, suggesting that viromes in these environments have a tremendous undiscovered functional potential. Interestingly, 63 gene clusters encoding beta-lactamases were found, suggesting that viromes include ARGs. Most of these ARGs were discovered on surfaces like skin, doorknobs, and bollards, indicating that viromes may play a role in spreading antibiotic resistance inbuilt settings (Du et al., 2023).

## 4 Discovery of NCLDV-host interaction (NHI)

Targeting a wide variety of eukaryotic species, including protists, mammals, and algae, NCLDVs are a varied group of viruses. These viruses may replicate in the host cell's cytoplasm and nucleus, which sets them apart from other viruses. Their genome sizes range from hundreds of kilobases to over a megabase. (Kim et al., 2023a; Mirzakhanyan and Gershon, 2020). These viruses have evolved complicated interactions with their eukaryotic hosts in terrestrial and marine environments. Many eukaryotes, such as coral species, phytoplankton, and zooplankton, are susceptible to infection. These interactions influence processes like primary productivity and nutrient cycling and play a crucial role in determining the dynamics of ecosystems (Meng et al., 2021; Messyasz et al., 2020).

**Fig. 6:**
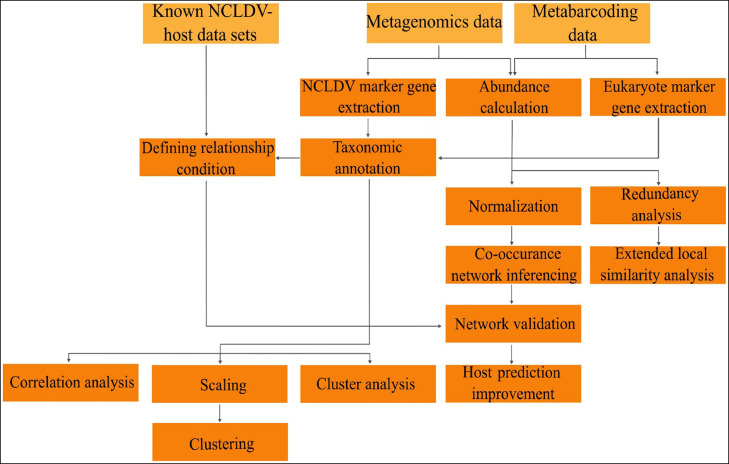
Workflow of NHI discovery.

Discovery of NHI via co-occurrence analysis utilizing metabarcoding and metagenomics data developed by Meng et al. proceeds through two different routes, including sequence of analysis based on abundance calculation and network creation via organism's marker genes (*polB* for NCLDV and V9 for protists) in parallel (Fig. 6) (Meng et al., 2021). After evaluation of marker genes extraction via the placer phylogenetic placement method, these data must be aggregated with discovered NHI datasets from previous works to define relationship conditions for NDLDVs and their hosts. These achieved network-based data are essential for network validation of data, which is achieved through abundance calculation. The second route of this method is launched by abundance calculation and normalizing the calculated data. The next step is inferencing the co-occurrence network, which is implemented by the FlashWeave tool, followed by a network validation procedure that is based on several procedures, including a confusion matrix defined by known NCLDV-host information, BLASTP searches of PolB included environmental sample sequences against NCLDV reference database, the positive likelihood ratio for assessing prediction accuracy, and false discovery rate for evaluating false positives. This procedure ended with improving host prediction, which was implemented by the Taxon Interaction Mapper (Meng et al., 2021).

If researchers desire to determine ecological factors that impact NHI, redundancy analysis utilizing tools like CANOCO and performing extended local similarity analysis can be considered after abundance calculation. According to the recently published by Kim et al., it is also possible to determine NHI by performing a group of statistical analysis including Spearman's correlation analysis, scaling (in this case, non-metric multidimensional scaling utilizing PRIMER 6 tool), and clustering (in this case hierarchical agglomerative clustering with utilizing the group average method), and cluster analysis (in this case Bray–Curtis dissimilarity method and the group average) (Kim et al., 2023a).

Findings from previous studies indicated that NCLDVs could strongly correlate with some of their host lineages in a specific region—for instance, 24 eukaryotic lineages identified as NCLDV-associated taxonomic groups. Also, among the identified NCLDV family, *Phycodnaviridae, Iridoviridae*, and *Mimiviridae* families are more likely to interact with specific groups of eukaryotic hosts. A wide range of hosts was noted for *Mimiviridae*, of which 13 lineages—including algae, metazoans, and protozoans—were found. *Mimiviridae* is connected to the most enriched nodes in the metazoan taxonomic group, suggesting that this family may be important in controlling metazoan populations in aquatic settings. These findings are consistent with the significant correlations observed between five *Mimiviridae* taxonomic groups and nine eukaryotic plankton taxa in the Kim et al. (2023a) study datasets.

Moreover, *Phycodnaviridae* showed strong associations with six lineages, including Mamiellophyceae and Bacillariophyta. Notably, relationships between *Phycodnaviridae* and *Mimiviridae* and Rhodophyta and Ciliophora were found. This relationship suggests that these NCLDV families may cooperate or compete with one another to infect certain hosts. Furthermore, the fact that *Iridoviridae* were predominantly linked to Metazoa suggests that their host range is quite specialized (Meng et al., 2021). These results imply that host preferences vary across NCLDV families. *Phycodnaviridae* and *Iridoviridae* show more specialized relationships, but *Mimiviridae* has a wider host range. *Mimiviridae* and *Phycodnaviridae* are related to several eukaryotic lineages, including Rhodophyta, Ciliophora, and Dictyochophyceae, suggesting that these families share a host range. The complexity of NHI is highlighted by the variation in host range and specificity, underscoring the need for additional research to understand these interactions completely.

Considering environmental elements in NHI can provide an important context for potential environmental interactions. Significant connections were found in previous research on this topic between six usual NCLDV families, 14 typical NCLDV taxa, and 21 typical variables, such as environmental conditions and plankton phylum or class levels. The first and second axes of the redundancy analysis accounted for 43.4 % and 18.9 % of the variance, respectively. Furthermore, it was also found that there were salinity associations in April with four distinct NCLDV families, including *Mimiviridae, Pithoviridae, Asfarviridae*, and *Pandoraviridae*. Additionally, *Phycodnaviridae* and *Poxviridae* showed a substantial relationship with particular oceanographic parameters. There were relationships between these viruses and size-fractionated chlorophyll-a concentration. They were also connected to the temperature of the water, indicating that temperature may impact their activity and dispersal. Several NCLDV groups are also connected to the marine algae class Pelagophyceae (Kim et al., 2023a). These results emphasize the complex interactions between NCLDVs, possible hosts, and environmental factors, highlighting these viruses' intricate ecological roles in marine ecosystems.

These results emphasize the complex interactions between NCLDVs, possible hosts, and environmental factors, highlighting these viruses' intricate ecological roles in marine ecosystems. The techniques used offer an extensive framework for comprehending these relationships. A thorough investigation of virus-host relationships and environmental factors has been made possible using tools like Taxon Interaction Mapper and FlashWeave and statistical techniques like Spearman's correlation, multidimensional scaling, and clustering analysis. A multidisciplinary approach is required to fully understand the complexities of NCLDV-host interactions and how they affect ecosystem dynamics.

## 5 Discovery of virophage-NCLDV interaction (VNI)

Virophages and NCLDVs exhibit a complex and fascinating relationship in diverse environments, especially within marine ecosystems. Virophages, which are diminutive viruses, infect and multiply within the host cells of large viruses like NCLDVs (Yau et al., 2011). To achieve precious insight into the ecological and evolutionary consequences of virophages and their host interactions and expand knowledge of such relationships (Xu et al., 2020), it seems that the discovery of VNI can be considered one of the important scientific efforts of virus-host characterization in the realm of metagenomics.

**Fig. 7:**
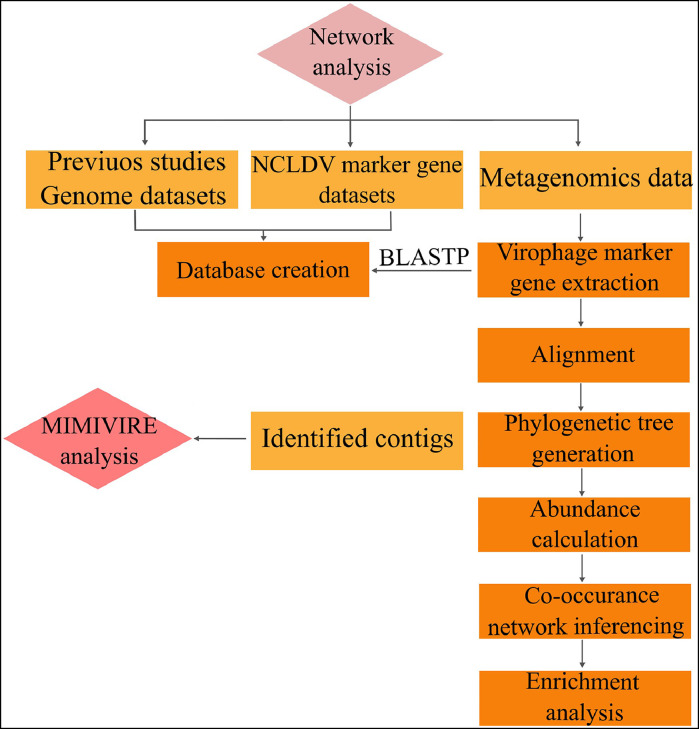
Workflow of VNI discovery.

Similar to the method described in Section 4.2.2. the first step in revealing possible VNI is to extract marker genes of virophages (*mcp* genes) (Fig. 7). In the next step, utilizing BLASTP against the created database, including genome datasets from previous studies and *polB* datasets, can result in the discovery of possible VNI. In order to ensure scrutinizing all possible VNI in the samples, other discovery methods can be performed in parallel with BLASTP. This process can be started by aligning achieved MCP amino acid sequences against databases. After phylogenetic analysis and abundance calculation of sequences, MCP and PolB sequences can be merged into matrices, and FlashWeave can conduct co-occurrence network inference. In the last step, data interpretation must be performed using a proper statistical analysis and adjusting method (in this case, the Fisher exact test and Benjamin-Hochberg adjusting method) (Meng et al., 2021).

Using identified virophage and NCLDV contigs to uncover possible VNI based on NCLDV anti-virophage defense systems is also possible. One of the simplest methods to discover VNI is a prediction of VNI based on the MIMIVIRE system connection. The Mimivirus Virophage Recognition Element, or the MIMIVIRE system, enables the mimivirus to identify and protect itself against virophages (Levasseur et al., 2016). Discovery of VNI through this system involves detecting the connection of virophage and mimivirus contigs when they share at least one sequence of 24–30 nucleotides at 100 % identity in both genomes, as well as at least single repeated subset of approximately 18 nucleotides within the same mimivirus gene (Paez-Espino et al., 2019).

VNI is not restricted to the interactions between virophages and NCLDVs; eukaryotic hosts are also important in this scenario. Advanced metagenomics and virophage association investigations shed light on the intricate and noteworthy interactions between NCLDVs and their eukaryotic hosts. A deeper understanding of the ecological roles and interactions of these viral organisms is made possible by this multifaceted approach (Xu et al., 2020). The goal of recent research has been to comprehend any possible connections between virophages and NCLDVs. For example, investigations revealed a ferritin-like superfamily protein common to GOVv18 and GOVLV1, 2, 3, 8, and 9, as well as a 2OG-FeII Oxy superfamily protein common to GOVLV6, GOVLV7, and GOVv34. These virophages and huge algal viruses cluster into different clades according to phylogenetic trees built with these proteins, indicating tight evolutionary connections. Furthermore, clustering tendencies resembling those seen between known virophage-NCLDV couples, including sputnik-mamavirus, were revealed by codon usage frequency analysis. These findings support the hypothesis of interactions between GOV virophages and big algal viruses, exemplified by the potential interaction network among Haptolina alga, GOVv18, and GOVLV1 (Wu et al., 2023).

Using FlashWeave to analyze data, researchers have identified 535 associations, most of which are positive, across different size fractions. *Mimiviridae* exhibited the greatest number of virophage relationships, underscoring its important function in interactions amongst virophages. A phylogenetic analysis identified Three main virophage clades, showing several relationships with NCLDVs. Significant correlations between particular virophage clades and NCLDV families, particularly between *Phycodnaviridae* and *Mimiviridae*, have been revealed using the Fisher exact test (Meng et al., 2021). A study by Paez-Espino et al. looked for shared amino acid patterns between large viruses and anticipated virophage sequences. This led to the identification of multiple linkages, most involving *Mimiviridae*, which is comparable to the studies that were previously stated. A new interaction has been found: a virophage linked to a large virus from the asfar-faustovirus cluster. These co-infecting virophages and massive viruses have been predicted to infect eukaryotic hosts, and these predictions have shown relationships with marine protists such *as Bigelowiella natans* and *Alexandrium tamarense*, showing the complex host-virus interactions in marine ecosystems (Paez-Espino et al., 2019).

The intricate interactions between virophages and NCLDVs in marine environments provide important insights into ecological and evolutionary processes. Sophisticated metagenomic techniques, such as co-occurrence network inference and BLASTP analysis, have revealed the critical function of eukaryotic hosts and found several VNI. The *Mimiviridae* family is noteworthy for having the greatest number of virophage relationships. New interactions have also been found, including those involving the asfar-faustovirus cluster. These results highlight the need for more investigation to clarify the various host-virus dynamics and their wider implications.

## 6 Discovery of polinton-like viruses-host interactions (PHI)

Polinton-like viruses (PLVs) are a varied class of small dsDNA viruses that can infect various eukaryotic organisms, commonly marine algae (Roitman et al., 2023; Roux et al., 2023). These viruses are believed to be the predecessors of many eukaryotic dsDNA viruses and exhibit similarities to polintons and virophages (Krupovic et al., 2016). PLVs are commonly found in freshwater environments, which can be significantly more prevalent in the virus size fraction than the microbial fraction. Studies have shown these viruses are among the most abundant in freshwater lake virus metagenomes, making them suitable targets for virus-host interaction studies (Bellas and Sommaruga, 2021).

**Fig. 8:**
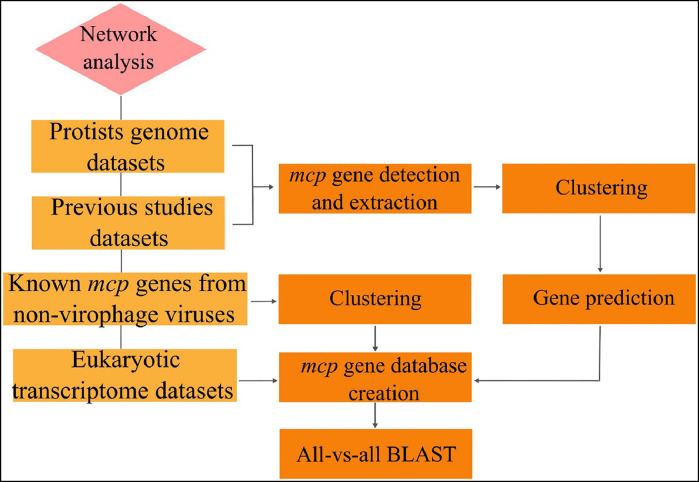
Workflow of PHI discovery.

Like the abovementioned virus-host interactions, PHI can be discovered by network analysis (Fig. 8). This procedure is based on performing all-vs-all BLAST, generating a similarity network from the created *mcp* gene database. The creation of this database is based on merging achieved *mcp* gene datasets from previous studies or datasets (Bellas et al., 2023; Bellas and Sommaruga, 2021).

Previous studies' results revealed that MCPs related to *Paulinella micropora* fell into at least three major superclusters, confirming the presence of diverse viral elements within these genomes. The known adintoviruses, vertebrate-associated Maverick-Polintons, and original Maverick-Polintons largely formed a large supercluster, subdivided into Group I and II Polintons, with exceptions like *mcp* genes from *Guillardia theta* and *Cnidaria*. This analysis highlighted that the original Maverick-Polintons are distinct viruses primarily associated with metazoans, with notable exceptions (Bellas et al., 2023).

Additionally, Maveriviricetes *mcp* genes formed a single cluster, underscoring their large divergence from other groups. Specific PLV groups, such as the gossevirus GKS1 group, exhibited distinct clustering patterns, often aligning with specific host types like oomycetes and chrysophytes. Furthermore, several MCP clusters from multiple eukaryotic lineages indicated instances of horizontal gene transfer during their evolutionary history (Bellas et al., 2023). A wide host range of PLVs is also discussed in Bellas et al. previous work. In the mentioned study, it is discovered that three *mcp* genes belonged to the PgVV group of PLV, associated with chlorophyte and haptophyte hosts, including *Polyblepharides amylifera, Prymnesium parvum*, and *Chrysochromulina rotalis*. This analysis demonstrated that PLVs are associated with a wide range of eukaryotic lineages, with some groups showing specific host associations, such as the gossevirus group with Stramenopiles and the PgVV group with haptophytes (Bellas and Sommaruga, 2021).

Considering everything, the discovery of PHI through network analysis has provided substantial insights into the diversity and evolutionary relationships of PLVs with their eukaryotic hosts. By utilizing all-vs-all BLAST to generate similarity networks from comprehensive *mcp* gene databases, studies have revealed the presence of diverse viral elements within eukaryotic genomes, such as *P. micropora* and various metazoans. The clustering patterns identified distinct groups like the gossevirus GKS1, highlighting specific associations with hosts like oomycetes and chrysophytes and instances of horizontal gene transfer. This method underscores the broad host range of PLVs, including chlorophytes and haptophytes, reinforcing their ecological significance and prevalence in aquatic environments.

## 7 Conclusion

The study of viral interaction and discovery has been completely transformed by metagenomics, which is supported by cutting-edge NGS technologies and bioinformatics tools. Metagenomics provides a thorough and effective replacement for conventional laboratory techniques by enabling the quick identification and characterization of viruses from a variety of contexts. The enormous variety of viral genomes and the complexity of metagenomic data have presented obstacles for viral investigations, yet the incorporation of AI-based technologies has improved their accuracy and speed even more. Case studies from a variety of environments have shown how dynamically complex virus-host interactions have been, as well as how variable viral populations may appear. As metagenomic techniques advance, they have enormous promise to reveal the rich and varied world of viruses and offer important new understandings of their ecological functions and effects. Our understanding of viral communities will advance with the continuous development and use of cutting-edge bioinformatics pipelines and AI tools, which will ultimately lead to more effective management techniques for viral diseases and the exploitation of advantageous viral functionalities.

## CRediT authorship contribution statement

**Mohammadreza Rahimian:** Writing – review & editing, Writing – original draft, Visualization, Data curation. **Bahman Panahi:** Data curation, Conceptualization.

## Declaration of competing interest

The authors declare that there is not any competing of interest.

## Data Availability

No data was used for the research described in the article.
